# Introducing a Novel Model-Free Multivariable Adaptive Neural Network Controller for Square MIMO Systems

**DOI:** 10.3390/s22062089

**Published:** 2022-03-08

**Authors:** Arash Mehrafrooz, Fangpo He, Ali Lalbakhsh

**Affiliations:** 1 Macquarie University College, Macquarie University, Sydney, NSW 2113, Australia; arash.mehrafrooz@mq.edu.au; 2 Advanced Control Systems Research Group, College of Science and Engineering, Flinders University, Adelaide, SA 5042, Australia; fangpo.he@flinders.edu.au; 3 School of Engineering, Macquarie University, Ryde, NSW 2109, Australia; 4 School of Electrical & Data Engineering, University of Technology Sydney, Sydney, NSW 2007, Australia

**Keywords:** adaptive neural networks, model-free control, auto-tuning, error back-propagation, accumulated gradient, nonlinear systems, closed-loop stability

## Abstract

In this study, a novel Multivariable Adaptive Neural Network Controller (MANNC) is developed for coupled model-free n-input n-output systems. The learning algorithm of the proposed controller does not rely on the model of a system and uses only the history of the system inputs and outputs. The system is considered as a ‘black box’ with no pre-knowledge of its internal structure. By online monitoring and possessing the system inputs and outputs, the parameters of the controller are adjusted. Using the accumulated gradient of the system error along with the Lyapunov stability analysis, the weights’ adjustment convergence of the controller can be observed, and an optimal training number of the controller can be selected. The Lyapunov stability of the system is checked during the entire weight training process to enable the controller to handle any possible nonlinearities of the system. The effectiveness of the MANNC in controlling nonlinear square multiple-input multiple-output (MIMO) systems is demonstrated via three simulation studies covering the cases of a time-invariant nonlinear MIMO system, a time-variant nonlinear MIMO system, and a hybrid MIMO system, respectively. In each case, the performance of the MANNC is compared with that of a properly selected existing counterpart. Simulation results demonstrate that the proposed MANNC is capable of controlling various types of square MIMO systems with much improved performance over its existing counterpart. The unique properties of the MANNC will make it a suitable candidate for many industrial applications.

## 1. Introduction

Over the past few years, there has been a significant improvement in controlling Multiple-Input Multiple-Output (MIMO) systems using adaptive control methods [1]. Many proposed adaptive controllers rely on model-based approaches [2,3] where mathematical models of the respective dynamic systems must be identified either directly or indirectly in advance. For most industry applications in practice, however, there are significant challenges with MIMO systems’ model identifications. For instance, a predicted industrial plant model can be dynamically different from the true plant itself, due largely to the plant’s structural uncertainties, unmodeled nonlinearities, and time-varying natures [4,5,6]. In some cases, if a constraint of an actual system for any reason changes, in order to achieve the desired outcomes, the system’s model may need to be re-identified resulting in a redesigning of the corresponding controller [7]. Even at the circumstances where an exact model of a MIMO system could be identified, the controller designed for the predicted model of the system may still be subjected to conditional variations both internal and external to the system [8]. Due to these practical problems associated with model-based approaches, many existing adaptive control schemes are seen to be impractical or limited in controlling real industrial MIMO plants. In contrast to model-based approaches, model-free approaches [9,10,11,12] can entirely omit the modeling phase of a system, thus significantly reducing the time required for the design and tuning of the system’s real-time controller. This will result in a faster and more precise control outcome [13,14,15]. Due to this consideration, model-free controllers are becoming more preferable than their corresponding model-based counterparts, especially for industrial MIMO control applications, where the modeling phase of a true plant can be time consuming and inaccurate. Among the recently introduced model-free control methods [13,15,16,17,18,19], the neural network technique stands out as a powerful and practical tool for controlling MIMO systems due mainly to its excellent capabilities in dealing with large volumes of data, estimating ambiguous relationships between a system’s inputs and outputs, and predicting future behaviors of a system. The application of neural networks is not limited to the MIMO systems and has been widely used in a range of engineering solutions, including substance characterizing [20,21,22,23], industrial engineering [24,25], microwave engineering [26,27], radiation accuracy [28,29], and measurement technologies [30,31,32,33]. Other nature-based algorithms, such as particle swarm [34,35,36], grey wolf [37,38,39], genetic algorithm [40], and ant colony [41], can be used in conjunction with the neural networks for further performance improvement.

Recently, by adding adaptive features to neural network schemes, adaptive neural networks have been seen in controlling MIMO systems successfully [42,43,44,45,46,47,48]. Many adaptive control methods based on neural networks have been introduced to Single-Input Single-Output (SISO) systems and further developed for Uncoupled Multiple-Input Multiple-Output (U-MIMO) systems [49,50,51,52]. In [45], a neural network controller and its associated learning rules are proposed, which can be successfully applied to Single-Input Multi-Output (SIMO) plants. This controller is a combination of several SISO controllers cascaded together and cannot be further developed for coupled MIMO systems. Since in general control problems are more challenging if cross-couplings among the various inputs and outputs of a MIMO system exist, model-free control of coupled MIMO systems has become an active area of research with a growing number of publications [53,54,55,56,57,58,59]. Despite some improvements, however, neural network-based controllers have not been extensively used in industrial model-free control systems due to the following apparent deficiencies [60]:

- During the weight training process of the neural networks, the controlled systems can become unstable;
- It is not always clear when to stop the weight training process;
- A long training time for the weights can be unsatisfactory for the speed of the control systems;
- The traditional activation functions employed in the neural networks may not be suitable for control purposes;
- The common error back-propagation learning algorithm uses only the last two consecutive samples of the outputs in discrete derivative functions and does not comply with the requirement of a proper model-free approach in which a full history of inputs and outputs must be used in order to generate an effective control action.

In order to overcome the above-listed deficiencies, in this study, a novel model-free Multivariable Adaptive Neural Network Controller (MANNC) is proposed for controlling coupled MIMO systems in which the following are true.

By using the constraint generated from the Lyapunov stability conditions at each step of the online weight training process, the overall control system stability can be guaranteed at all time; this eliminates the risk of the system been falling into its local minimum [61,62] and prevents the loss of the control speed due to conservative learning rate selections [15,18,47,62,63]:

- By constantly observing the accumulated errors and comparing them with their desired values, the controller can decide to stop the learning algorithm and lock the neural network weights at an optimal point; this ensures the convergence of the controller weight adjustments and provides a clear optimal number for the weight training steps;
- By choosing proper initial learning rates and dynamically changing them during the learning process according to the system stability criteria, the weight training speed can be significantly increased; this forms a clear comparison with and improvement over the traditional static learning rates [15,18,47,62,63];
- By designing specific activation functions that utilize typical proportional, integral, and derivative operations in the neural network structure of the controller, the proposed controller is simple and straightforward in its configuration; this makes the controller a potential candidate suitable for replacing classical PID controllers in industrial applications;
- By applying accumulated gradients in the error back-propagation algorithm and using new partial derivative estimations, the proposed method fully uses the history of the system outputs together with the current weights to produce the outputs of the controller (the inputs of the system) for the next step. This new learning method significantly reduces the overshoot and settling time of the system by minimizing the summation of errors of the system outputs in each step rather than using only the last two consecutive samples of the system outputs as its traditional counterparts do [61,64,65,66,67], and allows for the closed-loop system to achieve its best control performance with a minimum number of weight training steps.

It is anticipated that, being truly model-free, the proposed MANNC can generate adequate control actions over a wide range of operating conditions. Additionally, by using a new cross-coupling network structure, the proposed controller is expected to be able to control strongly-coupled MIMO systems effectively. It should be mentioned that, according to the design to be presented in this paper, the proposed MANNC will only be applicable for square (n × n) MIMO systems. If an industrial MIMO system has different numbers of inputs and outputs, by squaring up (adding) or squaring down (removing) the inputs (i.e., the manipulated variables) or the outputs (i.e., the controlled variables), the given non-square MIMO system can be rewritten as a corresponding square MIMO system [68] to which the proposed MANNC will be applicable.

To reveal the proposed MANNC, the rest of this paper is organized as follows. In Section 2, the structure and matrix representation of the new neural network-based adaptive controller are introduced. In Section 3, the new learning method of the neural network based on the error back-propagation algorithm is described. The closed-loop system stability is analyzed in Section 4. In Section 5, the controlled system and the introduced method are specified for SISO systems. Section 6 demonstrates the validation results via simulation studies where the proposed method is seen to control three chosen nonlinear MIMO systems of a drum-boiler, a time-variant nonlinear MIMO system, and a hybrid system without the use of their respective models. Conclusions in relation to the design are drawn in Section 7.

## 2. Multivariable Adaptive Neural Network Controller

### 2.1. Closed-Loop Structure of MANNC

As previously proposed in [18] by the authors, the outputs of three types of neurons: P-type, I-type, and D-type, in the discrete form can be respectively expressed as:

(1) $$o_{P}\left(k\right)=net_{P}\left(k\right)$$

(2) $$o_{I}\left(k\right)=o_{I}\left(k-1\right)+net_{I}\left(k\right)$$

(3) $$o_{D}\left(k\right)=net_{D}\left(k\right)-net_{D}\left(k-1\right)$$

where $o_{X}\left(k\right)$ and $net_{X}\left(k\right)$ represent the X-type neuron’s output and input at the $k^{\mathrm{th}}$ sample time, respectively. In this study, for stability concerns, further constraints to the neurons’ activation functions of Equations (1)–(3) are applied as follows:

(4) $$o_{P}\left(k\right)=\{\begin{array}{c} 1\;net_{P}\left(k\right)>1\\ net_{P}\left(k\right)-1\leq net_{P}\left(k\right)\leq 1\\ -1\;net_{P}\left(k\right)<-1 \end{array}$$

(5) $$o_{I}\left(k\right)=\{\begin{array}{c} 1\;net_{I}\left(k\right)>1\\ o_{I}\left(k-1\right)+net_{I}\left(k\right)-1\leq net_{I}\left(k\right)\leq 1\\ -1\;net_{I}\left(k\right)<-1 \end{array}$$

(6) $$o_{D}\left(k\right)=\{\begin{array}{c} 1\;net_{D}\left(k\right)>1\\ net_{D}\left(k\right)-net_{D}\left(k-1\right)-1\leq net_{D}\left(k\right)\leq 1\\ -1\;net_{D}\left(k\right)<-1 \end{array}$$

**Figure 1:**
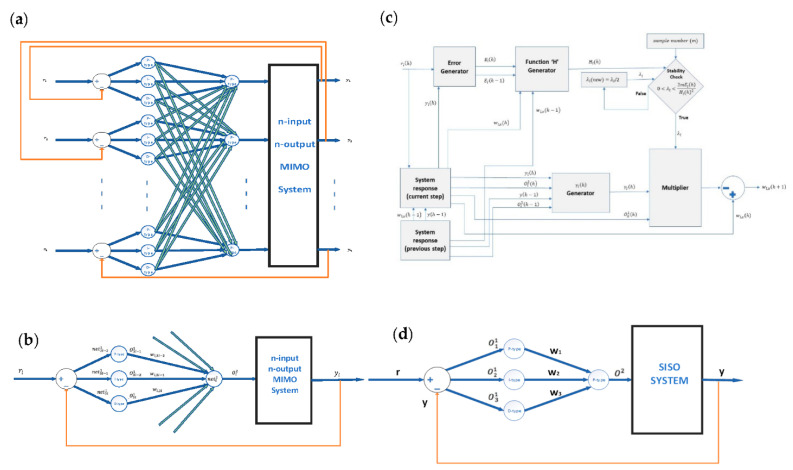
(**a**) Structure of the proposed MANN, (**b**) S-MANNC structure, (**c**) flowchart of real-time simultaneous stability criteria check and weight adjustment algorithm for MANNC, and (**d**) applying MANNC to a SISO system.

In [18], a new Adaptive Neural Network Controller (ANNC) structure was proposed. The ANNC contained six neurons and three layers. In the input layer, there are two P-type neurons, which perform the distribution of the inputs in the constructed neural network. In the hidden layer, there are three neurons each being a P-type, an I-type, and a D-type, respectively; the P-neuron compares the desired output with the actual output; the I-neuron provides the necessary action to eliminate the steady-state error; the D-neuron predicts the future behavior of the error. In the output layer, there is a P-type neuron, which performs the summation of the PID functionalities of the neuron. In this paper, a new Multivariable Adaptive Neural Network Controller (MANNC) is proposed in Figure 1a as a closed-loop MIMO controller applicable to coupled square (n×n) multivariable systems.

Assuming that there are strong cross-coupling between the n inputs and n outputs of a MIMO system of concern, the proposed MANNC is designed using a n−3n−n neural network structure. In this structure, the error for each output (i.e., the difference between each desired output $\left(r_{i}\right)$ and its corresponding actual output $\left(y_{i}\right)$ (i=1, 2, …, n)) is generated, and the generated errors propagate to the two layers, the hidden layer and the output layer of the MANNC network. In the hidden layer, there are 3n neurons including clusters of P-type, I-type, and D-type neurons that are repeated consecutively. In the output layer, there are n P-type neurons that form the outputs of the MANNC, i.e., the inputs of the n×n MIMO system. There are $3n^{2}$ weights in the output layer that are associated with the hidden-layer neurons and decide the impact of each neuron of the hidden layer on the generation of the inputs applied to the MIMO system.

### 2.2. Structure of Sub-MANNC (S-MANNC)

To be able to deal with the cross-couplings of an n×n MIMO system of concern, the proposed MANNC structure in Figure 1a is further decomposed into n-parallel sub-controllers each named as a Sub-MANNC (S-MANNC) and illustrated in Figure 1b. The neural network associated with the $l^{\mathrm{th}}$ S-MANNC with input $r_{l}$ and output $y_{l}$ (l=1, 2, …, n) is designed to have two layers (hidden layer and output layer) and four neurons; each neuron is able to be connected with the neurons of the other S-MANNCs in order to account for the cross-coupling effects of the underlying MIMO system. In the hidden layer of the $l^{\mathrm{th}}$ S-MANNC, there are three neurons each being a P-type, an I-type, and a D-type, respectively. The P-neuron amplifies the difference between the desired output and the actual output, the I-neuron provides the necessary action to eliminate the steady-state error, and the D-neuron predicts the future behavior of the error. In the output layer of the $l^{\mathrm{th}}$ S-MANNC, there is a P-type neuron, which performs the summation of the PID functionalities of the hidden-layer neurons. This neuron accumulates the outputs of the hidden layer and forms the control command applied to the $l^{\mathrm{th}}$ output of the n×n MIMO system. Due to the fact the MANNC will be used for controlling coupled multivariable systems, the output neuron of each S-MANNC has ‘n’ number of inputs to be able to produce each control command by considering all the desired outputs and actual outputs. The inputs of the P-type, I-type, and D-type neurons ($net_{3l-2}^{1}$, $net_{3l-1}^{1}$, and $net_{3l}^{1}$) and the outputs of these neurons ($O_{3l-2}^{1}$, $O_{3l-1}^{1}$, and $O_{3l}^{1}$) are related together by the activation functions of the neurons represented in Equations (4)–(6). There are three weights ($w_{1,3l-2}$, $w_{1,3l-1}$, and $w_{1,3l}$) in the output layer of each S-MANNC, relating to the P-type, I-type, and D-type neurons in the hidden layer.

### 2.3. Matrix Representation

The matrix representation of a closed-loop square MIMO system using the proposed MANNC Figure 1a and S-MANNC (Figure 1b) is derived as follows. Let $O_{l}^{2}$ and $y_{l}$ be, respectively, the $l^{\mathrm{th}}$ input and the $l^{\mathrm{th}}$ output of the system, where 1≤ l ≤n, and let $G_{ij}$ be the transfer function relating the $j^{\mathrm{th}}$-component of the $i^{\mathrm{th}}$ output ($y_{i}$) to the $j^{\mathrm{th}}$ input ($O_{j}^{2}$), where 1≤ i ≤n and 1≤ j ≤n. The vectors and matrices associated with Figure 1b are named in Table 1 and are defined as follows.

(7) $$Y=Y_{n\times 1}={\left[\begin{array}{ccc} y_{1} & y_{2} & \begin{array}{cccc} \cdots & y_{l} & \cdots & y_{n} \end{array} \end{array}\right]}_{1\times n}{}^{T}$$

(8) $$O_{}^{2}=O_{n\times 1}^{2}={\left[\begin{array}{ccc} O_{1}^{2} & O_{2}^{2} & \begin{array}{cccc} \cdots & O_{l}^{2} & \cdots & O_{n}^{2}{}_{n} \end{array} \end{array}\right]}_{1\times n}{}^{T}$$

(9) $$G=G_{n\times n}={\left[\begin{array}{ccc} \begin{array}{c} G_{11}\\ G_{21}\\ \vdots \end{array} & \begin{array}{c} G_{12}\\ G_{22}\\ \vdots \end{array} & \begin{array}{cccc} \cdots & G_{1l} & \cdots & G_{1n}\\ \cdots & G_{2l} & \cdots & G_{2n}\\ & & & \vdots \end{array}\\ \begin{array}{c} G_{l1}\\ \vdots \\ G_{n1} \end{array} & \begin{array}{c} G_{l2}\\ \vdots \\ G_{n2} \end{array} & \begin{array}{cccc} \cdots & G_{ll} & \cdots & G_{ln}\\ & \vdots & & \;\vdots \\ \cdots & G_{nl} & \cdots & G_{nn} \end{array} \end{array}\right]}_{n\times n}$$

(10) $$net_{}^{2}=net_{n\times 1}^{2}={\left[\begin{array}{ccc} net_{1}^{2} & net_{2}^{2} & \begin{array}{cccc} \cdots & net_{l}^{2} & \cdots & net_{n}^{2} \end{array} \end{array}\right]}_{1\times n}{}^{T}$$

(11) W=Wn×3n=[w1,1w2,1⋮w1,2w2,2⋮w1,3⋯w1,3l−2w1,3l−1w2,3⋯w2,3l−2w2,3l−1   ⋮wl,1⋮wn,1wl,2⋮wn,2wl,3⋯wl,3l−2wl,3l−1    ⋮wn,3⋯wn,3l−2wn,3l−1 w1,3lw2,3l⋮⋯w1,3n−2w1,3n−1w1,3n⋯w2,3n−2w2,3n−1w2,3n   ⋮   wl,3l⋮wn,3l⋯wl,3n−2wl,3n−1wl,3n ⋮⋮⋮ ⋯ wn,3n−2wn,3n−1 wn,3n]n×3n

(12) $$O_{}^{1}=O_{3n\times 1}^{1}={\left[\begin{array}{ccc} O_{1}^{1} & O_{2}^{1} & \begin{array}{ccc} O_{3}^{1} & \cdots & O_{3n}^{1} \end{array} \end{array}\right]}_{1\times 3n}{}^{T}$$

(13) $$P=P_{3n\times 3n}={\left[\begin{array}{ccc} \begin{array}{ccc} 1 & 0 & 0\\ 0 & D^{-1} & 0\\ 0 & 0 & D \end{array} & \cdots & 0\\ \vdots & \ddots & \vdots \\ 0 & \cdots & \begin{array}{ccc} 1 & 0 & 0\\ 0 & D^{-1} & 0\\ 0 & 0 & D \end{array} \end{array}\right]}_{3n\times 3n}$$

(14) $$net_{}^{1}=net_{3n\times 1}^{1}={\left[\begin{array}{ccc} net_{1}^{1} & net_{2}^{1} & \begin{array}{ccc} net_{3}^{1} & \cdots & net_{3n}^{1} \end{array} \end{array}\right]}_{3n\times 1}{}^{T}$$

(15) $$R_{}^{\left(3\right)}=R_{3n\times 1}^{\left(3\right)}={\left[\begin{array}{ccc} r_{1} & r_{1} & \begin{array}{cccccccc} r_{1} & r_{2} & r_{2} & r_{2} & \cdots & y_{n} & y_{n} & y_{n} \end{array} \end{array}\right]}_{1\times 3n}{}^{T}$$

(16) $$Y_{}^{\left(3\right)}=Y_{3n\times 1}^{\left(3\right)}={\left[\begin{array}{ccc} y_{1} & y_{1} & \begin{array}{cccccccc} y_{1} & y_{2} & y_{2} & y_{2} & \cdots & y_{n} & y_{n} & y_{n} \end{array} \end{array}\right]}_{1\times 3n}{}^{T}$$

(17) $$I_{}^{\left(3\right)}=I_{3n\times n}^{\left(3\right)}={\left[\begin{array}{ccc} \begin{array}{c} 1\\ 1\\ 1 \end{array} & \cdots & 0\\ \vdots & \ddots & \vdots \\ 0 & \cdots & \begin{array}{c} 1\\ 1\\ 1 \end{array} \end{array}\right]}_{3n\times n}$$

(18) $$R=R_{n\times 1}={\left[\begin{array}{ccc} r_{1} & r_{2} & \begin{array}{ccc} r_{3} & \cdots & r_{n} \end{array} \end{array}\right]}_{1\times n}{}^{T}$$

Considering that the system at each calculation step can be identified as being linear or been linearized around an operation point, the relationship between the inputs and outputs of the system will be as follows:

(19) $$Y_{n\times 1}=G_{n\times n}O_{n\times 1}^{2}$$

Since $net_{l}^{2}$ is the $l^{th}$ input of the P-type neuron in the output layer, one has the following:

(20) $$O_{n\times 1}^{2}=net_{n\times 1}^{2}$$

where the relationship between the inputs and outputs in the output layer of the neural networks can be expressed as follows:

(21) $$net_{n\times 1}^{2}=W_{n\times 3n}^{2}O_{3n\times 1}^{1}$$

As the hidden layer has clusters of P-type, I-type, and D-type neurons, the proportional, integral, and derivative operators (1, $D^{-1}$, and D) are considered, respectively, in the matrix form of the activation function. Thus, one has the following:

(22) $$O_{3n\times 1}^{1}=P_{3n\times 3n}net_{3n\times 1}^{1}$$

where the inputs of the hidden layer are defined as the differences between the desired outputs and the actual outputs and are expressed as follows:

(23) $$net_{3n\times 1}^{1}=R_{3n\times 1}^{3}-Y_{3n\times 1}^{3}$$

By having

(24) $$R_{3n\times 1}^{\left(3\right)}=I_{3n\times n}^{\left(3\right)}\times R_{n\times 1}$$

and

(25) $$Y_{3n\times 1}^{\left(3\right)}=I_{3n\times n}^{\left(3\right)}\times Y_{n\times 1}$$

and using Equations (19)–(25), one obtains

(26) $$Y=GWP(R^{\left(3\right)}-Y^{\left(3\right)})\;=\;GWP\left(I^{\left(3\right)}R-I^{\left(3\right)}Y\right)$$

Hence, the system’s outputs can be derived as follows:

(27) $$Y=GWPI^{\left(3\right)}R{\left(I+GWPI^{\left(3\right)}\right)}^{-1}$$

where $\left|I+GWPI^{\left(3\right)}\right|\neq 0$.

Additionally, by having

(28) $$Y=GWPI^{\left(3\right)}\left(R-Y\right)$$

one has

(29) $$G^{-1}Y=WPI^{\left(3\right)}\left(R-Y\right)$$

where |G|≠0.

Since $PI^{\left(3\right)}\left(R-Y\right)$ is a non-square matrix and P is symmetrical, from

(30) $$G^{-1}Y{\left\{PI^{\left(3\right)}\left(R-Y\right)\right\}}^{T}=WPI^{\left(3\right)}\left(R-Y\right){\left\{PI^{\left(3\right)}\left(R-Y\right)\right\}}^{T}$$

one derives

(31) $$W=G^{-1}Y{\left(R-Y\right)}^{T}I^{\left(3\right)}{}^{T}P{\left[PI^{\left(3\right)}\left(R-Y\right){\left(R-Y\right)}^{T}I^{\left(3\right)}{}^{T}P\right]}^{-1}$$

where W is expressed as a function of the desired outputs and the actual outputs, and $\left|PI^{\left(3\right)}\left(R-Y\right){\left(R-Y\right)}^{T}I^{\left(3\right)}{}^{T}P\right|\neq 0$ and |W|≠0.

The system can then be identified as follows:

(32) $$G=Y{\left(R-Y\right)}^{T}I^{\left(3\right)}{}^{T}P{\left[PI^{\left(3\right)}\left(R-Y\right){\left(R-Y\right)}^{T}I^{\left(3\right)}{}^{T}P\right]}^{-1}W^{-1}$$

where |W|≠0.

It should be pointed out that, although Equations (19)–(32) are derived under the assumption that the system is linear or can be linearized around an operating point, they can potentially be used for nonlinear systems where the nonlinearities of the systems can be approximated by piece-wise linear systems whose time-varying nature can account for the nonlinearities of the systems satisfactorily. It should also be pointed out that if any of the non-singularity conditions for matrices $\left(I+GWPI^{\left(3\right)}\right)$, (G), $\left(PI^{\left(3\right)}\left(R-Y\right){\left(R-Y\right)}^{T}I^{\left(3\right)}{}^{T}P\right)$, and (W) could not be met, the selected weights (matrix W) would not be acceptable and would be re-updated until all these matrices become non-singular.

## 3. Learning Algorithm

To achieve a precise control effect for a square MIMO system, the neural network weights of the MANNC are adjusted using the principle of the multi-step error back-propagation algorithm described in [69]. Back-propagation is one of the standard methods of training artificial neural networks. This method efficiently computes the weights of one layer at a time using the delta rule. It helps calculate the gradient of a loss function with respect to all the weights in the network. The algorithm is to find the set of weights that minimizes the error by the steepest descent direction calculated for the loss function versus the present weights. The weights will be updated along the steepest descent direction, and the error is reduced in every step [70,71]. The choice of this algorithm is based on the considerations that the system is treated as a “black-box” and the controller will be designed based on a model-free approach. In this study, instead of using merely the current gradient of the system error as the literature does for SISO systems [47,62,63], an accumulated gradient of the system error for the last m samples is proposed to be used to achieve a more precise control performance. The proposed method minimizes the sum of the square accumulated gradient of the error for each system output in each learning step, where the error is taken as the difference between the desired output $r_{l}\left(k\right)$ (i.e., the system setpoint) and the actual output $y_{l}\left(k\right)$. The Euclidean norm E is defined for calculating the quadratic cost function of the system for the system error. The power of two in this expression makes the error of each output positive, so that larger errors will be more significant than the smaller errors. The cost function for the $l^{\mathrm{th}}$ S-MANNC in Figure 1b is then defined as follows:

(33) $$E_{l}\left(h\right)=\frac{1}{2}{\left(\overset{m}{\underset{k=1}{\sum }}\left(r_{l}\left[k\right]-y_{l}\left(h\right)\left[k\right]\right)\right)}^{2}$$

where $E_{l}\left(h\right)$ is the error of the $l^{\mathrm{th}}$ output in the $h^{\mathrm{th}}$ step number of the learning algorithm and m is the required number of discrete samples in the system output and the set-point. By increasing m, the system output will be compared more accurately with the setpoint. However, a large value of m may decrease the adjustment speed of the controller and become undesirable when the speed of the control system (often a critical requirement for a real-time industrial system) is of concern. Therefore, a reasonable value of m must be used to make a necessary trade-off between the desired accuracy and the required speed of the control system. The total cost function of the system (J), which is the sum of n errors by considering all outputs, is written as follows:

(34) $$J\left(h\right)=\overset{n}{\underset{l=1}{\sum }}E_{l}\left(h\right)=\frac{1}{2}\overset{n}{\underset{l=1}{\sum }}{\left(\overset{m}{\underset{k=1}{\sum }}\left(r_{l}\left[k\right]-y_{l}\left(h\right)\left[k\right]\right)\right)}^{2}$$

Using the accumulated gradient of the system error, a learning algorithm must be designed to minimize the defined cost function and to bring the system outputs as close as possible to the desired outputs. According to the principle of the error back-propagation learning algorithm, the output layer’s weights must be adjusted so that in each step they move slightly in the opposite direction of the gradient of the cost function. This is to ensure that the cost function will be decreasing step by step. The weights of the output layer will therefore be adjusted based on the following learning rule:

(35) $$w_{l,x}\left(h+1\right)=w_{l,x}\left(h\right)-\lambda_{l}\frac{\partial J\left(h\right)}{\partial w_{l,x}}$$

where 1≤x≤3l and 1≤l≤n, h is the step number of the learning algorithm, and $w_{l,x}\left(h\right)$ and $w_{l,x}\left(h+1\right)$ are the weights of the output layer in the current and the following steps, respectively; $\lambda_{l}$ is the learning rate which decides how fast the cost is changing and, in particular, determines the weight adjustment speed. The gradient of the error subject to each weight is required to be calculated. Using partial derivatives, one has

(36) $$\frac{\partial J}{\partial w_{l,x}}=\frac{\partial J}{\partial E_{l}}\frac{\partial E_{l}}{\partial y_{l}}\frac{\partial y_{l}}{\partial O_{l}^{2}}\frac{\partial O_{l}^{2}}{\partial net_{l}^{2}}\frac{\partial net_{l}^{2}}{\partial w_{l,x}}$$

where

(37) $$\frac{\partial J}{\partial E_{l}}=1$$

(38) $$\frac{\partial E_{l}}{\partial y_{l}}=\overset{m}{\underset{k=1}{\sum }}(y_{l}\left(h\right)\left[k\right]-r_{l}\left(h\right)\left[k\right])$$

(39) $$\frac{\partial y}{\partial O_{l}^{2}}≅\frac{\sum_{k=1}^{m}\left(y\left(h\right)\left[k\right]\right)-\sum_{k=1}^{m}\left(y\left(h-1\right)\left[k\right]\right)}{\sum_{k=1}^{m}\left(O_{l}^{2}\left(h\right)\left[k\right]\right)-\sum_{k=1}^{m}\left(O_{l}^{2}\left(h-1\right)\left[k\right]\right)}$$

(40) $$\frac{\partial O_{l}^{2}}{\partial net_{l}^{2}}=1$$

Because the output neuron is a ‘P-type’ neuron, one writes

(41) $$\frac{\partial net_{l}^{2}}{\partial w_{l,x}}=\overset{m}{\underset{k=1}{\sum }}\left(O_{x}^{1}\left(h\right)\left[k\right]\right)$$

Substituting Equations (37)–(41) into (36), one obtains

(42) $$\frac{\partial J}{\partial w_{l,x}}≅1\times \overset{m}{\underset{k=1}{\sum }}(y_{l}\left(h\right)\left[k\right]-r_{l}\left(h\right)\left[k\right])\frac{\sum_{k=1}^{m}\left(y\left(h\right)\left[k\right]\right)-\sum_{k=1}^{m}\left(y\left(h-1\right)\left[k\right]\right)}{\sum_{k=1}^{m}\left(O_{l}^{2}\left(h\right)\left[k\right]\right)-\sum_{k=1}^{m}\left(O_{l}^{2}\left(h-1\right)\left[k\right]\right)}O_{x}^{1}\left(h\right)\left[k\right]$$

Defining $\gamma_{l}\left(k\right)$ as

(43) $$\gamma_{l}\left(h\right)=\overset{m}{\underset{k=1}{\sum }}(y_{l}\left(h\right)\left[k\right]-r_{l}\left(h\right)\left[k\right])\frac{\sum_{k=1}^{m}\left(y\left(h\right)\left[k\right]\right)-\sum_{k=1}^{m}\left(y\left(h-1\right)\left[k\right]\right)}{\sum_{k=1}^{m}\left(O_{l}^{2}\left(h\right)\left[k\right]\right)-\sum_{k=1}^{m}\left(O_{l}^{2}\left(h-1\right)\left[k\right]\right)}$$

the output layer weight adjustment rule can thus be derived as

(44) $$w_{l,x}\left(h+1\right)=w_{l,x}\left(h\right)-\lambda_{l}[\gamma_{l}\left(h\right)\overset{m}{\underset{k=1}{\sum }}\left(O_{x}^{1}\left(h\right)\left[k\right]\right)]$$

The resulting weight adjustment algorithm is summarized in Table 2.

Using the described learning method, all weights are simultaneously tuned, and their values at the current step, together with all the current inputs and all the current outputs, are used to create the outputs for the weights’ training at the next step. This online dynamic learning feature of the proposed method (i.e., applying the accumulated gradient and the accumulated errors) makes it more suitable for MIMO systems than its existing counterparts that are mainly applicable for SISO systems [61,62,72]. The neural network structure presented for the MANNC is designed for a general n-input n-output system with cross-couplings among all its inputs and outputs. This structure can be simplified for systems with less cross-coupling to reduce the number of the associated weights and, consequently, to increase the learning speed.

## 4. Stability Analysis

It is well known that when a new control method is proposed, it is necessary to investigate the stability condition of the resultant closed-loop system in order to ensure the achievement of the desired control outcomes. For an unconstrained control system, the system stability can be defined using Bounded-Input Bounded-Output (BIBO) stability criteria. While the eigenvalue analysis concept based on the BIBO stability criteria can be used to investigate the stability condition of a linear system, it can not be applied to nonlinear systems. Instead, the Lyapunov stability analysis concept becomes a useful tool for nonlinear systems. As the proposed model-free MANNC method will inherently result in a nonlinear closed-loop control system, its closed-loop stability investigation will need to be carried out using the Lyapunov stability analysis concept. Although, unlike a linear system, the stability of a nonlinear system may not need to be global as the system can have multiple equilibrium points and limit cycles, and global asymptotic stability is sought in this study for the proposed MANNC to ensure its satisfactory closed-loop performance over a wide range of operating points. This will make the controller more suitable for use in industrial applications. According to the Lyapunov global asymptotic stability theorem, for a defined function V(x), if:

(i) V(0)=0
(ii) (For all x≠0, V(x)>0 (i.e., *V* is positive definite)
(iii) For all x≠0, ΔV(x)<0

Then every trajectory of $\dot{X}=f\left(X\right)$ will converge to zero as *t*→∞ and the system will be globally asymptotically stable.

Consider a model-free MANNC control system whose Lyapunov function for each of its outputs is defined as follows:

(45) $$V_{l}\left(h\right)=E_{l}\left(h\right)$$

where $E_{l}\left(h\right)$ is the cost function related to the $h^{th}$ step of the learning algorithm. One can write

(46) $$\Delta V_{l}\left(h\right)={\left(E_{l}\left(h\right)+\Delta E_{l}\left(h\right)\right)}^{2}-{\left(E_{l}\left(h\right)\right)}^{2}=2E_{l}\left(h\right)\Delta E_{l}\left(h\right)+{\left(\Delta E_{l}\left(h\right)\right)}^{2}$$

and:

(47) $$\Delta E_{l}\left(h\right)≅\Delta w_{l,x}\left(h\right)\overset{m}{\underset{k=1}{\sum }}\frac{\partial E_{l}\left(h\right)\left[k\right]}{\partial w_{l,x}}$$

From Equation (46), one has

(48) $$\Delta w_{l,x}\left(h\right)=-\frac{\lambda_{l}}{m}\overset{m}{\underset{k=1}{\sum }}\frac{\partial J}{\partial w_{l,x}}$$

where

(49) $$\frac{\partial J}{\partial w_{l,x}}=\frac{\partial J}{\partial E_{l}\left(h\right)}\frac{\partial E_{l}\left(h\right)}{\partial w_{l,x}}=\frac{\partial E_{l}\left(h\right)}{\partial w_{l,x}}$$

Substitute Equation (49) into Equation (47), respectively, and one derives

(50) $$\Delta w_{l,x}\left(h\right)=-\frac{\lambda_{l}}{m}\overset{m}{\underset{k=1}{\sum }}\frac{\partial E_{l}\left(h\right)\left[k\right]}{\partial w_{l,x}}$$

and

(51) $$\Delta E_{l}\left(h\right)≅-\frac{\lambda_{l}}{m}{\left(\overset{m}{\underset{k=1}{\sum }}\frac{\partial E_{l}\left(h\right)\left[k\right]}{\partial w_{l,x}}\right)}^{2}$$

Hence, Equation (46) can be expressed as

(52) $$\Delta V\left(h\right)≅-\frac{2\lambda_{l}E_{l}\left(h\right)}{m}{\left(\overset{m}{\underset{k=1}{\sum }}\frac{\partial E_{l}\left(h\right)\left[k\right]}{\partial w_{l,x}}\right)}^{2}+\frac{\lambda_{l}{}^{2}}{m^{2}}{\left(\overset{m}{\underset{k=1}{\sum }}\frac{\partial E_{l}\left(h\right)\left[k\right]}{\partial w_{l,x}}\right)}^{4}$$

Define

(53) $$H_{l}\left(h\right)=\overset{m}{\underset{k=1}{\sum }}\frac{\partial E_{l}\left(h\right)\left[k\right]}{\partial w_{l,x}}\;≅\overset{m}{\underset{k=1}{\sum }}\frac{\Delta E_{l}\left(h\right)\left[k\right]}{\Delta w_{l,x}\left(h\right)}$$

The condition for ΔV(h)<0 will yield the following constraint on the selection of the learning rate $\lambda_{l}$ defined in Equation (44):

(54) $$0<\lambda_{l}<\frac{2mE_{l}\left(h\right)}{H_{l}{\left(h\right)}^{2}}$$

If the above constraint is satisfied at each training step, the system will be globally asymptotically stable during the entire training process. This indicates that Equation (54) must be checked in a real-time and simultaneous fashion at each training step during the operation of the weight adjustment algorithm. The flowchart of the whole real-time weight training process, including both the learning algorithm (Table 2) and the stability criteria check in Equation (54), is illustrated in Figure 1c.

## 5. Specifying MANNC to Control SISO Systems

The proposed MANNC can be used in a SISO environment where the number of inputs and outputs of the controller is chosen as one, i.e., n=1. The resultant SISO closed-loop system is shown in Figure 1d. Considering Figure 1d and assuming a linear and time-invariant estimation for the system’s SISO transfer function, one has

(55) $$Y=G\left(s\right)O^{2}=G\left(s\right)(w_{1}O_{1}^{1}\left(s\right)+w_{2}D^{-1}O_{2}^{1}\left(s\right)+w_{3}DO_{3}^{1}\left(s\right))$$

where

(56) $$O_{1}^{1}\left(s\right)=O_{2}^{1}\left(s\right)=O_{3}^{1}\left(s\right)=R\left(s\right)-Y\left(s\right)$$

Then,

(57) $$Y\left(s\right)=G\left(s\right)(w_{1}+w_{2}s^{-1}+w_{3}s)\left(R\left(s\right)-Y\left(s\right)\right)$$

and

(58) $$\frac{Y\left(s\right)}{R\left(s\right)}=\frac{G\left(s\right)(w_{1}+w_{2}s^{-1}+w_{3}s)}{1+G\left(s\right)(w_{1}+w_{2}s^{-1}+w_{3}s)}$$

If $w_{1}$, $w_{2}$, and $w_{3}$ are taken as the coefficients of a classical proportional, integral, and derivative controller, i.e., $K_{P}$, $K_{I}$, and $K_{D}$, respectively, the resultant control system will perform similar to an auto-tune SISO PID control system whose closed-loop transfer function is expressed as follows:

(59) $$T\left(s\right)=\frac{G\left(s\right)(K_{P}+K_{I}s^{-1}+K_{D}s)}{1+G\left(s\right)(K_{P}+K_{I}s^{-1}+K_{D}s)}$$

The weight learning algorithm illustrated in Table 2 can be customized for SISO system applications and described in Table 3 where λ is the learning rate.

Following the Lyapunov stability analysis described in Equations (45)–(52) and redefining Equation (53) as follows:

(60) $$H\left(h\right)=\overset{m}{\underset{k=1}{\sum }}\frac{\partial E\left(h\right)\left[k\right]}{\partial w_{l,x}}\;≅\overset{m}{\underset{k=1}{\sum }}\frac{\Delta E\left(h\right)\left[k\right]}{\Delta w_{x}\left(h\right)}$$

the condition for ΔV(h)<0 yields

(61) $$0<\lambda <\frac{2mE\left(h\right)}{H{\left(h\right)}^{2}}$$

## 6. Simulation Results

Over the recent years, various computational approaches, such as finite element, finite difference time domain, finite-difference frequency-domain, and Lyapunov stability analysis have been successfully applied to a variety of control systems, including photonic crystals [73,74,75,76,77,78], high-frequency passive components [79,80,81,82,83,84,85,86,87], electromagnetic devices [88,89,90,91,92,93,94], fluid mechanical engineering [95,96], and MIMO control systems [97,98]. Simulation studies using MATLAB are carried out to evaluate the performances of the proposed MANNC in tracking setpoints, reducing unwanted overshoots or undershoots, and securing the global stability of a closed-loop system during the system’s entire control process. The structure of the MANNC proposed in Section 2, the dynamic neural network algorithm developed in Section 3, and the stability criteria checking condition discussed in Section 4 are used in three simulation cases, each presenting a type of square MIMO systems. They are a time-invariant nonlinear system, a time-variant nonlinear system, and a hybrid system.

### 6.1. Case 1: Application of MANNC on a Time-Invariant Nonlinear Square MIMO System

**Figure 2:**
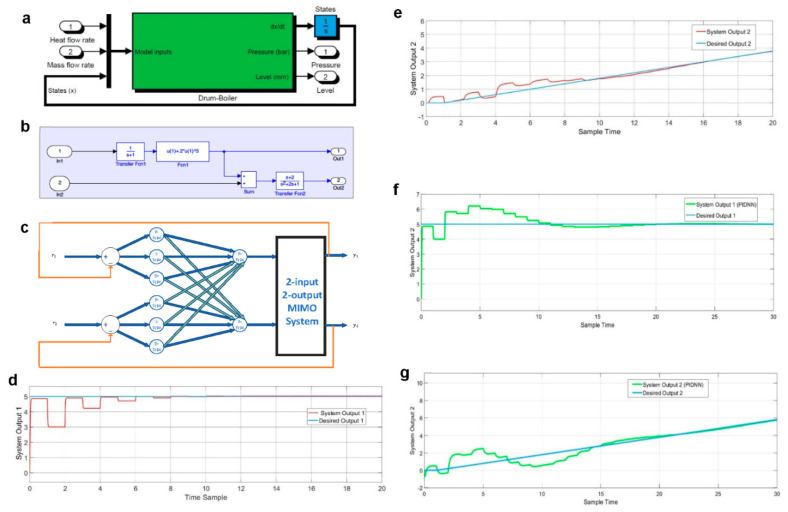
(**a**) Two-input two-output drum-boiler system, (**b**) two-input two-output nonlinear drum-boiler model, and (**c**) two-input two-output system controlled by MANNC. (**d**) Case 1: output one and desired output one (MANNC), (**e**) case 1: output two and desired output two (MANNC), (**f**) case 1: output one and setpoint one (PIDNN), and (**g**) case 1: output two and setpoint two (PIDNN).

In this case, a drum-boiler plant (Figure 2a), which is a generic nonlinear time-invariant coupled two-input two-output system with heat flow rate and mass flow rate as inputs and pressure and level as outputs, is chosen. The nonlinearities of the plant cause the system dynamic characteristics to vary with operating conditions. In addition, cross-couplings and parameter variations of the plant makes it a challenging case to control. For years, constructing a nonlinear controller directly from the original nonlinear model of the drum-boiler has been a general approach to use in order to improve the system performance in compensating the plant’s nonlinearities. In this study, however, the proposed model-free MANNC method is used in which the nonlinear model of the drum-boiler is not required by the controller design process and only the inputs and outputs of the plant are used for the construction of the controller. The performances of the MANNC in improving the system setpoint tracking time and reducing undesirable overshoots are compared with those of the best and foremost existing neural network method reported in the literature.

The nonlinear state equations describing the relationships between the inputs and outputs of the drum-boiler system of Figure 2a are written as follows:

(62) $$\dot{x_{1}}\left(t\right)=-x_{1}\left(t\right)+u_{1}\left(t\right)$$

(63) $$y_{1}\left(t\right)=x_{1}\left(t\right)+2x_{1}^{3}\left(t\right)$$

(64) $$x_{2}\left(t\right)=y_{1}\left(t\right)+u_{2}\left(t\right)$$

(65) $$\ddot{y_{2}}\left(t\right)+2\dot{y_{2}}\left(t\right)+y_{2}\left(t\right)=\dot{x_{2}}\left(t\right)+2x_{2}\left(t\right)$$

where “dot” denotes time derivative, $u_{1}\left(t\right)$ and $u_{2}\left(t\right)$ are respectively the heat flow rate and mass flow rate as the system inputs, $y_{1}\left(t\right)$ and $y_{2}\left(t\right)$ are respectively the pressure and level as the system outputs, and $x_{1}\left(t\right)$ and $x_{2}\left(t\right)$ are the state variables. The simulation block diagram of the given nonlinear system is presented in Figure 2b, where box ‘Fcn1’ produces the highly nonlinear relationship between the inputs and outputs of the system. Due to the fact that Input 1 affects both Output 1 and Output 2, the system is also a cross-coupled MIMO system and, thus, will not be able to be controlled by multiple ANNC controllers introduced in [99], nor by any other SISO or non-coupled MIMO counterparts.

Figure 2c demonstrates the implementation of the proposed MANNC in the two-input two-output system. In this neural network, 12 weights in the hidden layer must be trained. The following desired outputs are selected for the drum-boiler system.

− $r_{1}\left(t\right)=5.0u\left(t-1\right)$ where u(t) is the standard unit step function.
− $r_{2}\left(t\right)=0.2r\left(t-1\right)$ where r(t) is the standard unit ramp function.

These desired outputs represent a scenario in which the pressure will tend to reach 5 bars and the level will increase following a smooth ramp function. The ramp function is chosen to be the setpoint for Output 2 in order to make the MANNC control more challenging. The objective is to find suitable values for the weights of the output layer so as to force the outputs, $y_{1}$ and $y_{2}$, to follow the desired setpoints, $r_{1}$ and $r_{2}$, respectively.

To apply the MANNC to the drum-boiler system, all initial weights are set to 1, the learning rates are set to 0.1 ($\lambda_{1}=\lambda_{2}=0.1$), and the number of samples is set to 40 (m=40). After repeating, simultaneously, the online learning algorithm (Table 2) and the Lyapunov stability criteria check (Figure 1c, 20 times), the final weight values of the output layer of the MANNC neural network are obtained and reported in Table 4.

During the running of the weight training algorithm, the control system remains stable all the time as $\lambda_{1}$ and $\lambda_{2}$ are kept unchanged. If the control system becomes unstable at a training step, the learning rates will be reduced, and the learning algorithm will be continued with a lower training speed that is dictated by the new learning rates. Applying the final adjusted weights (Table 4) to the control system, Figure 2d shows that Output 1 tracks the desired step output properly with a zero overshoot. This zero-overshoot effect is most desirable for many industrial control systems, as unwanted overshoots during setpoint changes can bring devastated results to the industrial plants. For example, when filling a hazardous liquid tank in a water plant, overshoots can result in overflows if the setpoint is close to the tank’s height [99]. Figure 2e demonstrates that Output 2 tracks the desired ramp output properly, and in less than nine samples the output can reach the desired level with a less than 5% error.

To demonstrate the anticipated highly improved performance of the MANNC over its existing adaptive counterparts, a most recent neural network controller (named PIDNN) introduced in [61] is chosen for the drum-boiler system. The PIDNN uses a learning algorithm that is based on two consecutive time-samples. The weights of the PIDNN are set after running the learning algorithm 20 times—the same condition upon which the weights of the MANNC are obtained. The comparison results are given in Figure 2f,g. It is seen that using the PIDNN method, Output 1 presents a 22% undesirable overshoot, and Output 2 exhibits a slower setpoint tracking with larger fluctuations.

The comparison results between the MANNC and the PIDNN control effects with an equal number of trainings are given in Table 5. It is evident that, compared to the PIDNN control system, the MANNC control system can significantly reduce the required training time (by 59%), achieve a zero overshoot (100% reduction), and reach the 5% and 2% error bands in much shorter times (47% and 50% faster for Output 1, and 47% and 36% faster for Output 2, respectively). These results demonstrate a superior performance of the MANNC over its existing counterpart. In particular, Output 1 under MANNC control exhibits a deadbeat-like response with minimum rise time, minimum settling time, no overshoot, and no steady-state error, comparable with an optimal closed-loop response. This remarkable result is attributed to the MANNC strategy that uses the accumulated error and the response of the system in consecutive learning steps rather than in consecutive sample times.

**Figure 3:**
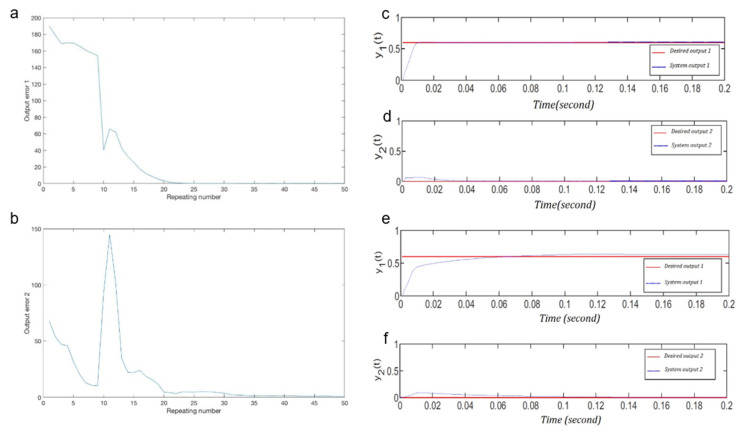
(**a**) Case 1: error value for Output 1 versus the repeating number and (**b**) case 1: error value for Output 2 versus the repeating number; (**c**) case 2: Output 1 and desired Output 1 by MANNC, (**d**) case 2: Output 2 and desired Output 2 by MANNC, (**e**) case 2: Output 1 and desired Output 1 by PIDNN, and (**f**) case 2: Output 2 and desired Output 2 by PIDNN.

Figure 3a,b represent, respectively, the accumulated error versus the iteration number of the MANNC weight learning algorithm for Output 1 and Output 2. The errors shown in these figures are the differences between the actual outputs and the desired outputs. It is observed that, with the MANNC, the magnitudes of the errors generally decrease as the number of iterations in the weight learning algorithm increases. This is evidence for the convergence of the proposed learning algorithm in Section 3 and is viewed as a significant result that demonstrates the suitable performance of the proposed MANNC for the considered coupled two-input two-output nonlinear system. As shown in these figures, with 35 trainings, the errors are reasonably low, and the values of the weights can be locked in at this point. By continuing the weight adjustment algorithm up to 50 times, the steady-state errors for both outputs become nearly zero. In general, choosing the optimal training number in this method depends on the particular application in which the controller is employed, and implies a trade-off between the control speed and the control performance of the closed-loop system. It is possible to pre-define a desired accumulated error, so that when the actual error reaches to that value, the training process stops and the weights become locked until the next change in the system happens (e.g., a change in the model of the system and/or a change in any of the setpoints).

### 6.2. Case 2: Application of MANNC on a Time-Variant Nonlinear MIMO System

In this case, a discrete time, highly nonlinear, time-variant two-input two-output coupled system is chosen to test the suitability and performance of the MANNC for time-variant MIMO systems. The chosen system is expressed as:

(66) $$y_{1}\left(k+1\right)=\frac{1}{y_{1}{}^{2}\left(k\right)+1}\left(0.8y_{1}\left(k\right)+v_{1}\left(k-2\right)+0.2v_{2}\left(k-3\right)\right)$$

(67) $$y_{2}\left(k+1\right)=\frac{1}{y_{2}{}^{2}\left(k\right)+1}\left(0.9y_{2}\left(k\right)+0.3v_{1}\left(k-3\right)+v_{2}\left(k-2\right)\right)$$

where $y_{1}\left(k\right)$ and $y_{2}\left(k\right)$ are the system outputs, and $v_{1}\left(k\right)$ and $v_{2}\left(k\right)$ are the system inputs. The desire outputs are selected as $r_{1}\left(k\right)=0.6$ and $r_{2}\left(k\right)=0$.

By applying, simultaneously, the online learning algorithm (Table 2) and the stability criteria check (Figure 1c) 50 times, the final weight values of the output layer of the MANNC neural network are obtained and reported in Table 6.

**Figure 4:**
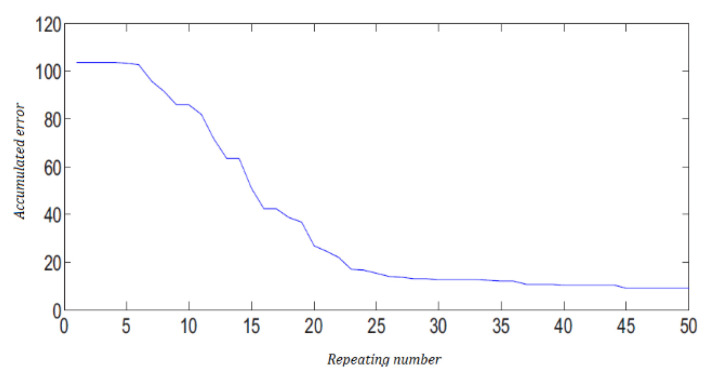
Case 2: accumulated error vs repeating number for MANNC.

The simulation results of the final adjusted weights of the MANNC are shown in Figure 3c,d. In order to compare the MANNC results with those of a properly selected existing counterpart, the PIDNN introduced in [61] is applied to the same system. The system outputs under the PIDNN control are shown in Figure 3e,f. It is demonstrated that both outputs of the MANNC control system track the desired outputs faster than those of the PIDNN control system. In addition, a zero overshoot in Output 1 using the MANNC is achieved, which is a significant result as this is not achievable by using the PIDNN. Decreasing the accumulated error by increasing the number of iterations, as shown in Figure 4, demonstrates the convergence of the proposed learning algorithm for the MANNC evidently. Table 7 presents the performance comparison between the MANNC controller and the PIDNN controller with an equal number of trainings. As can be seen, the MANNC control results outperform the PIDNN control results in all aspects.

### 6.3. Case 3: Application of MANNC on a Hybrid System

**Figure 5:**
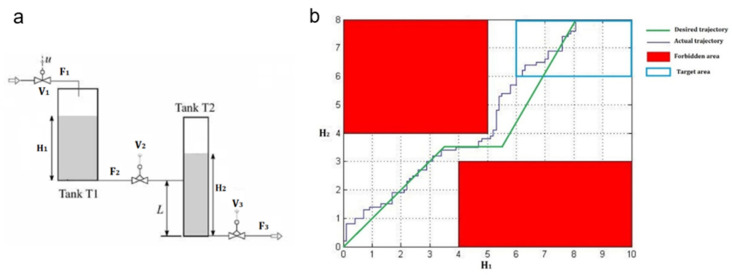
(**a**) Two-tank hybrid system, and (**b**) actual and desired state trajectories.

Since the MANNC has been designed as a universal controller for black-box square MIMO systems, in this case, the performance of this controller is tested on a hybrid system. By definition, a hybrid system is a dynamic system that switches between continuous states and, thus, involves both continuous and discrete behaviors. Due to sudden changes in system dynamics at the time of switching between two states, conventional control methods are usually unsuccessful for hybrid systems. In this study, the two-tank plant shown in Figure 5a was selected to test the control performance of the MANNC. The plant consists of two tanks, where tank T1 is filled by flow $F_{1}$ through a fully open valve $V_{1}$. The liquid is transferred from tank T1 into tank T2 via a connecting pipe. Valve $V_{2}$ is an on-off valve, which is either fully open or fully closed to adjust flow $F_{2}$ discretely. Similarly, flow $F_{3}$ is adjusted by another on-off valve $V_{3}$. When the behavior of the given plant is modeled, it must be considered that the liquid levels, $H_{1}$ and $H_{2}$, respectively, for both tanks will change separately when $H_{2}$ crosses level L. At $H_{2}=H_{1}+L$ the direction of flow through the inter-connecting pipe is reversed. Hence, for $F_{2}$, two dynamics must be modeled as:

(68) $$F_{2}=\{\begin{array}{c} k_{1}\cdot V_{2}\cdot \sqrt{\left(H_{1}-H_{2}+L\right)}\;if\;H_{2}>L\;\\ \;k_{1}\cdot V_{2}\cdot \sqrt{\left(H_{1}\right)}\;if\;H_{2}\leq L\; \end{array}$$

and

(69) $${\dot{H}}_{1}=(F_{1}-F_{2})/k_{3}$$

(70) $${\dot{H}}_{2}=(F_{2}-k_{2}\cdot V_{3}\cdot \sqrt{H_{2}})/k_{4}$$

where $k_{1}$, $k_{2}$, $k_{3}$, and $k_{4}$ are constants depending on characteristic coefficients of the pipes and the cross-section areas of the tanks, and $V_{2}$ and $V_{3}$ represent the Boolean values in which “1” and “0” signify, respectively, a fully open valve and a fully closed valve.

It is evident that the given plant is a nonlinear hybrid system with two inputs ($V_{2}\;\mathrm{and}\;V_{3}$) and two outputs ($H_{1}\;\mathrm{and}\;H_{2}$). The control problem is defined as: using the on-off control valves $V_{2}$ and $V_{3}$, the liquid heights ($H_{1}$ and $H_{2}$) in tanks should be derived from an initial state of $H_{0}=\left(0.01,0.01\right)$ to a target area of $R_{T}$ with the following conditions:

(71) H∈[0,10]×[0,8]

(72) $$H_{f}\in R_{T}=\left[6,10\right]\times \left[6,8\right]$$

where $H_{f}$ represents the final desired heights located in the target area ($R_{T}$). In addition, the forbidden areas, $R_{F1}$ and $R_{F2}$, are considered as:

(73) $$H\notin R_{F1}=\left[0,5\right]\times \left[4,8\right]$$

(74) $$H\notin R_{F2}=\left[4,10\right]\times \left[0,3\right]$$

**Figure 6:**
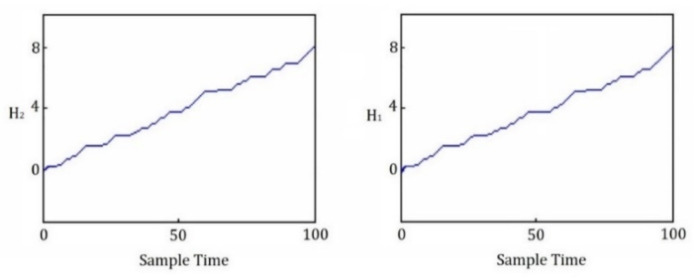
Height of liquid in tank T1 and tank T2.

The desired liquid heights that lead the actual liquid heights to the target area are defined as:

(75) $$R_{H1}=0.08r\left(t\right)$$

(76) $$R_{H2}=\{\begin{array}{c} R_{H1}\;if\;R_{H1}<3.5\\ 3.5\;if\;3.5\leq R_{H1}<5.5\\ 1.8R_{H1}-6.4\;if\;5.5\leq R_{H1}\leq 8 \end{array}$$

where r(t) is a standard ramp function. The final desired heights for both tanks in this simulation are selected as 8 meters i.e., $H_{f}=\left[8,8\right]$. After repeating, simultaneously, the online learning algorithm (Table 2) and the stability criteria check 20 times, the state trajectories of the MANNC control system for the liquid heights were achieved and are shown in Figure 5b, where the forbidden areas and the desired track are also illustrated. It is clear that, by using the MANNC in this complex nonlinear hybrid control problem, the liquid height trajectory can pass the narrow zone between the forbidden areas successfully and eventually reach the target area. This is achieved due to the powerful and fast setpoint tracking property of the proposed method that can cope with changes in the system dynamics. In addition, the liquid heights of the two tanks versus time during this process are shown in Figure 6. The above results are achieved using the final weight values of the output layer of the MANNC neural network, shown in Table 8. Since the final desired liquid heights can be selected as any points in the target area, simulation studies are carried out for several points in this area and the accumulated errors are computed in a range of 285 to 310. Regardless of the location of the selected target point in the target area, the accumulated error is restricted, and the convergence is evidently achieved. This justifies the suitability and adequacy of the proposed MANNC in controlling the highly complex hybrid system.

## 7. Conclusions

A model-free MANNC capable of satisfactorily controlling nonlinear square MIMO systems with significant cross-couplings within a short period of time is presented in this paper. The MANNC uses a new auto-tune dynamic online learning algorithm with accumulated error back-propagation for the proposed neural network structure, and effectively tunes its weights to achieve the desirable control outcomes. The learning algorithm is integrated with the Lyapunov stability criteria while running and applied to the control system. The effectiveness of the proposed MANNC is validated via simulation studies on typical time-invariant and time-variant square MIMO systems. When compared with the best representative of existing counterparts (i.e., the PIDNN) for applications to both time-invariant and time-variant systems, the MANNC in the time domain is seen to provide less overshoot, less settling time, less accumulated errors, and faster setpoint tracking. The MANNC can be effectively used for several types of square MIMO control systems in industrial applications by selecting an appropriate number of samples, especially when overshoots in outputs are undesirable and fast setpoint tracking is critical. The simulation results for controlling a complex and challenging MIMO hybrid system demonstrate the superior performance of the MANNC when it deals with significant changes in the system dynamics. As a future study, adding more layers to the neural network structure of the MANNC can be considered in order to further improve the control performance, especially for highly nonlinear plants.

## Figures and Tables

**Table 1:** Matrices defined for MANNC.

$Y_{n\times 1}$	$R_{n\times 1}$	$O_{n\times 1}^{2}$	$G_{n\times n}$
System’s outputs	System’s desired outputs	System’s inputs	System’s transfer matrix
$net_{n\times 1}^{2}$	$W_{n\times 3n}$	$O_{3n\times 1}^{1}$	$P_{3n\times 3n}$
Output layer’s inputs	Neural Network Weights	Neurons’ outputs	Activation functions
$net_{3n\times 1}^{1}$	$R_{3n\times 1}^{\left(3\right)}$	$Y_{3n\times 1}^{\left(3\right)}$	$I_{3n\times n}^{\left(3\right)}$
Hidden layer’s inputs	Triple desired outputs	Triple system’s outputs	Triple unit

**Table 2:** Weights adjustment learning algorithm.

1≤l≤n	$w_{l,x}\left(h+1\right)=w_{l,x}\left(h\right)-\lambda_{l}[\gamma_{l}\left(h\right)\sum_{k=1}^{m}\left(O_{x}^{1}\left(h\right)\left[k\right]\right)]$
1≤x≤3n	$\gamma_{l}\left(h\right)=\sum_{k=1}^{m}(y_{l}\left(h\right)\left[k\right]-r_{l}\left(h\right)\left[k\right])\frac{\sum_{k=1}^{m}\left(y\left(h\right)\left[k\right]\right)-\sum_{k=1}^{m}\left(y\left(h-1\right)\left[k\right]\right)}{\sum_{k=1}^{m}\left(O_{l}^{2}\left(h\right)\left[k\right]\right)-\sum_{k=1}^{m}\left(O_{l}^{2}\left(h-1\right)\left[k\right]\right)}$

**Table 3:** Specified weight learning algorithm for auto-tune classical PID controller.

$K_{P}\left(h+1\right)=K_{P}\left(h\right)-\lambda \gamma \left(h\right)\sum_{k=1}^{m}\left(O_{1}^{1}\left(h\right)\left[k\right]\right)]$
$K_{I}\left(h+1\right)=K_{I}\left(h\right)-\lambda \gamma \left(h\right)\sum_{k=1}^{m}\left(O_{2}^{1}\left(h\right)\left[k\right]\right)]$
$K_{D}\left(h+1\right)=K_{D}\left(h\right)-\lambda \gamma \left(h\right)\sum_{k=1}^{m}\left(O_{3}^{1}\left(h\right)\left[k\right]\right)]$
$\gamma \left(h\right)=\sum_{k=1}^{m}\left(y\left(h\right)\left[k\right]-r\left(h\right)\left[k\right]\right)\frac{\sum_{k=1}^{m}\left(y\left(h\right)\left[k\right]\right)-\sum_{k=1}^{m}\left(y\left(h-1\right)\left[k\right]\right)}{\sum_{k=1}^{m}\left(O^{2}\left(h\right)\left[k\right]\right)-\sum_{k=1}^{m}\left(O^{2}\left(h-1\right)\left[k\right]\right)}$

**Table 4:** Final weight values of the output layer.

$w_{l,x}$	x=1	x=2	x=3	x=4	x=5	x=6
l=1	10.23	0.33	6.93	−2.23	−2.13	−2.10
l=2	−1.35	3.59	1.36	3.22	3.24	1.94

**Table 5:** Case 1: MANNC vs. PIDNN.

Controller	Number of Trainings	Time of Training	Output 1 Overshoot	Output 1 Maximum Error Less than 5%	Output 1 Maximum Error Less than 2%	Output 2 Maximum Error Less than 5%	Output 2 Maximum Error Less than 2%
MANNC	20	1.48 s	0%	8 s.t. *	10 s.t.	9 s.t.	14 s.t.
PIDNN	20	3.67 s	22%	15 s.t.	20 s.t.	17 s.t.	22 s.t.

* s.t. stands for sample times.

**Table 6:** Final weight values of the output layer.

$w_{l,x}$	x=1	x=2	x=3	x=4	x=5	x=6
l=1	3.34	2.43	4.73	−5.12	−8.13	−2.19
l=2	−11.30	−4.44	−7.74	6.32	3.55	3.82

**Table 7:** Case 2: MANNC vs. PIDNN.

Controller	Number of Trainings	Time of Training	Output 1 Overshoot	Output 1 Maximum Error Less than 5%	Output 2 Maximum Error Less than 5%
MANNC	50	2.39 s	0%	0.01 s	0.05 s
PIDNN	50	3.99 s	22%	0.03 s	0.08 s

**Table 8:** Final weight values of the output layer.

$w_{l,x}$	x=1	x=2	x=3	x=4	x=5	x=6
l=1	3.1	1.21	2.43	−1.68	−4.34	−5.2
l=2	−0.13	−1.58	4.28	0.88	2.06	3.41

## Data Availability

Not applicable.
